# Identification of Safety Biomarkers for Autologous Blood Transfusion in Hepatocellular Carcinoma Patients

**DOI:** 10.1111/jcmm.70504

**Published:** 2025-04-07

**Authors:** Yong Cheng, Yue Wang, Xiao‐fang Zhou, Lai‐wei You, Xiao‐yi Xie, Hao Li, Mandi Wu, Jianrong Guo

**Affiliations:** ^1^ Department of Anesthesiology Gongli Hospital of Shanghai Pudong New Area Shanghai China

**Keywords:** autologous blood transfusion, hepatocellular carcinoma, safety biomarkers

## Abstract

Hepatocellular carcinoma (HCC) represents the predominant form of primary liver cancer, constituting 75%–85% of all liver cancer cases. Despite advances in understanding HCC mechanisms and treatment options, challenges remain and further research is needed to uncover new therapeutic targets and improve patient outcomes. intraoperative cell salvage (IOCS) is an important surgical method that minimises the necessity for transfusions of donor blood, improves haemodynamic stability and may enhance recovery. This study aims to identify safety biomarkers for autologous blood transfusion in HCC patients. We conducted a prospective case–control study on 80 HCC patients undergoing radical surgery. Blood and tumour tissues were collected for analysis. The control group provided blood directly from the surgical site without IOCS processing, while the experimental group used blood collected through the IOCS system. Dual‐Luciferase reporter gene assays, immunofluorescence, Western blot and qRT‐PCR techniques were employed to assess the expression of key proteins and microRNAs. Comparable demographic and baseline clinical characteristics were observed between groups. The experimental group showed significantly improved pathological features, with an increase in PTEN‐positive cells and upregulated protein expression of PTEN, mTOR, c‐Met and IGF1R. Additionally, miRNA levels (miRNA‐21, miRNA‐122, miRNA‐223, miRNA‐199a and miRNA‐375) were significantly reduced in the experimental group (*p* < 0.05), while mRNA levels for PTEN, mTOR, c‐Met, YAP1 and IGF1R were significantly upregulated (*p* < 0.05). IOCS has a positive impact on liver tissue pathology in HCC patients by reducing apoptosis and modulating key molecular pathways. These findings suggest that IOCS could be a valuable therapeutic strategy for HCC, potentially guiding future treatment approaches and improving patient outcomes.

**Abbreviations**
HCC: hepatocellular carcinoma
HE: haematoxylin and eosin
IOCS: intraoperative cell salvage
PI: propidium iodide
PVDF: polyvinylidene fluoride
TBST: Tris‐buffered saline containing Tween

## 1 Introduction

Autologous blood transfusion represents the most effective method for conserving blood during surgical operations [1]. Investigating the feasibility and safety of this practice for patients undergoing surgeries for malignant tumours addresses a pressing need in China's economic and social development [2]. Currently, allogeneic blood transfusion remains the predominant approach for these surgeries, but the persistent imbalance between blood supply and demand poses significant challenges, particularly for malignancies such as liver cancer, which account for over 30% of surgical blood usage [3]. Allogeneic transfusions are associated with immunosuppression in tumour patients, heightening risks of postoperative complications, including wound infections, tumour recurrence and metastasis [4]. While intraoperative cell salvage (IOCS) has gained traction in various surgical contexts, its application in malignant tumour surgeries is often considered taboo in China [5, 6]. This study seeks to explore the critical international issue of safely employing IOCS in liver cancer surgeries, aiming to challenge this taboo. A successful implementation of IOCS could potentially reduce the need for blood donors by 5 to 6 million annually, establishing a theoretical foundation for its safe use in malignant tumour surgeries. If realised, this research would mark a significant advancement in perioperative medicine, alleviating blood shortages and enhancing recovery and prognosis for tumour patients.

The identification of safety biomarkers is crucial for advancing the management of hepatocellular carcinoma (HCC) [7, 8], particularly in the context of autologous blood transfusion and IOCS [9, 10]. Despite the importance of this research, there is currently limited investigation into the safety biomarkers specific to HCC, creating an urgent need for studies in this area. The microRNAs miRNA‐21, miRNA‐122, miRNA‐223, miRNA‐199a and miRNA‐375 have shown potential as safety biomarkers for HCC [11, 12, 13, 14]. These microRNAs play significant roles in regulating gene expression and could provide valuable insights into the biological processes underlying HCC [15], thereby helping to predict patient outcomes and potential risks associated with autologous blood transfusion.

In addition to these microRNAs, other molecular targets such as PTEN, mTOR, c‐Met, YAP1 and IGF1R have been proposed as safety biomarkers in HCC [16, 17, 18, 19]. These proteins are involved in critical signalling pathways that influence cell growth, proliferation and survival, and their dysregulation is often associated with the progression of HCC. Investigating the expression levels and functional roles of these biomarkers could provide a more comprehensive understanding of the safety and efficacy of blood conservation strategies in HCC patients.

Given the limited research in this field, our study aims to fill this gap by exploring the roles of these biomarkers in HCC and assessing their potential to serve as indicators of safety in autologous blood transfusion practices. This research could pave the way for more targeted and effective treatment strategies, ultimately improving patient outcomes in HCC.

## 2 Materials and Methods

### 2.1 Patients

This research is a prospective case–control trial study, and the study involved 80 patients with HCC who underwent radical surgery at our hospital from January 2022 to December 2023. The patients were randomly divided into two groups: a control group and an experimental group, each consisting of 40 individuals. Blood samples were taken for analysis in the study. In the control group, blood samples obtained directly from the surgical site during the operation were analysed without any treatment and served as baseline data. In the experimental group, blood samples from the surgical area were retrieved using an IOCS device, then processed through a storage tank. This processing included centrifugation, separation and washing, followed by filtration through a leukocyte filter to eliminate potential tumour cells and impurities. All individuals involved in the study were made aware of its purpose and gave their written consent, in accordance with the approval from the hospital's Ethics Committee. The criteria for inclusion are outlined as follows: (1) Patients aged 20 years and above; (2) Confirmed liver cancer through preoperative diagnosis and postoperative histopathological examination; (3) Undergoing radical resection for liver cancer; (4) Substantial intraoperative blood loss requiring transfusion; (5) No anaemia before surgery; (6) Liver function categorised as Child‐Pugh grades A or B. The exclusion criteria are as follows: (1) Presence of concurrent heart or kidney failure; (2) Undergoing minor liver surface resection with minimal blood loss; (3) Concurrent coagulation disorders or haematological diseases; (4) Incomplete clinical data.

### 2.2 Transwell Assay

To evaluate the invasive properties of HCC cells, liver tumour cells were suspended in a serum‐free medium. A 200‐μL aliquot of this cell suspension was introduced into a transwell insert, which was pre‐coated with a thin layer of Corning Matrigel Matrix to mimic the extracellular matrix environment. The lower chamber of the transwell was filled with 600 μL of a nutrient‐enriched medium supplemented with 5% foetal bovine serum (FBS), providing essential nutrients and growth factors necessary for cell proliferation and migration. After a 24‐h incubation period at 37°C, the cells that had successfully migrated through the Matrigel matrix were collected and analysed using a Leica optical device to assess their invasive capabilities.

### 2.3 Immunofluorescence Assay

The immunofluorescence assay was initiated by applying stabilisation and infiltration techniques to liver tumour tissue samples to preserve cellular integrity and enhance antibody penetration. Following this, the tissues were immersed in a bath containing primary antibodies specific to the target proteins of interest. After allowing adequate time for incubation with the primary antibodies, the samples were promptly rinsed to remove any unbound antibodies, ensuring specificity in detection. Subsequently, the liver tumour tissue cells underwent a secondary incubation with fluorescently labelled secondary antibodies, which bind to the primary antibodies. This was followed by another wash to eliminate any remaining unbound secondary antibodies. Finally, the treated samples were examined using a fluorescence microscope, allowing for detailed analysis of the localisation and expression of the target proteins within the liver tumour tissue.

### 2.4 Flow Cytometry Assay

Liver tumour tissues were analysed for apoptosis levels using flow cytometry, following the manufacturer's instructions. After collection, the tissues were stained with Annexin V‐FITC and propidium iodide (PI) to differentiate between apoptotic and necrotic cells. The staining procedure was performed in a dark environment to prevent photobleaching of the fluorescent dyes. Once the staining was completed, the samples were processed through a flow cytometer, which allowed for the quantification of apoptosis levels across all experimental groups. The flow cytometry analysis provided a detailed assessment of cell viability and apoptotic cell populations based on the fluorescence emitted by the stained cells.

### 2.5 Western Blotting

Liver tumour tissue protein extracts were subjected to separation using 10% SDS‐PAGE, allowing for the resolution of protein components based on their molecular weight. After electrophoresis, the proteins were transferred onto polyvinylidene fluoride (PVDF) membranes for further examination. To remove any non‐specific binding, the membranes were washed with TBST (Tris‐buffered saline containing Tween 20). Primary antibodies specific to the target proteins, as well as actin (a loading control), were then applied to the membranes. These antibodies, sourced from Bioworld Technology Inc., China, were incubated overnight at 4°C to ensure optimal binding. After the incubation period, the membranes underwent thorough washing with TBST to remove any unbound primary antibodies. Secondary antibodies, also obtained from Bioworld Technology Inc., were added. The membranes were left to incubate at room temperature for 2 h. After this period, they were rinsed again with TBST to remove any remaining secondary antibodies. Protein bands were detected using an ECL chemiluminescence reagent, and the signals were subsequently analysed to assess the expression levels of the target proteins in the liver tumour tissue samples.

### 2.6 qRT‐PCR

Total RNA was isolated from liver tumour samples using TRIzol Reagent (Beyotime, Shanghai) according to the manufacturer's instructions to guarantee high‐quality RNA yield. Following this, the extracted mRNA was converted into complementary DNA (cDNA) using the mRNA Reverse‐Transcription Kit (Beyotime, Shanghai). To measure mRNA expression levels, quantitative PCR (qPCR) was conducted with the SYBR Green PCR Mix (Vazyme Biotech, Shanghai) on a Real‐Time PCR System. The relative expression levels of the target genes were determined using the 2 − ΔΔCt method, with normalisation to the housekeeping gene GAPDH. This process was repeated three times to ensure the reliability and precision of the results. The specific primers used for amplification were as follows (See Table 1):

**TABLE 1:** The specific primers used for amplification.

Gene	Forward primer	Reverse primer
miRNA‐21	TAGCTTATCAGACTGATGTTGA	CAACATCAGTCTGATAAGCTA
miRNA‐122	TGGAGTGTGACAATGGTGTTTG	CAAACACCATTGTCACACTCCA
miRNA‐223	TGTCACTGTGTCAGTTTGTC	GGGGTATTTGACAAACTGAC
miRNA‐199a	ACAGTAGTCTGCACATTGGTTA	TAACCAATGTGCAGACTACTGT
miRNA‐375	TTTGTTCGTTGCGGCTCGCGTGA	TCACGCGAGCCGAACGAACAAA
PTEN	TCTTGACCAATGGCTAAGTG	CTCTGACTGGGAATAGTTACTC
mTOR	ACGCTGTCATCCCTTTATC	CTTCTTCTTCTCCCTGTAGTC
c‐MET	ACTCCTACAACCCGAATAC	CCTCATCATCAGCGTTATC
YAP1	CCAGCACAGCAAATTCTC	AGCTGCTCATGCTTAGTC
IGF1R	TAAGAACCAGTGGCGAAAG	ACGCAACCAGTCATCTAAG
GAPDH	CACCCACTCCTCCACCTTTG	CCACCACCCTGTTGCTGTAG

### 2.7 Dual‐Luciferase Reporter Assay

To validate the direct interaction between miRNA‐21 and PTEN, as well as the effect of miRNA‐199a on mTOR, a dual‐luciferase reporter assay was performed. For this experiment, cells were co‐transfected with PTEN wild‐type (PTEN‐WT) or PTEN mutant (PTEN‐MUT) reporter constructs, or with mTOR 3′‐UTR wild‐type (3′‐UTR‐WT) or mutant (3′‐UTR‐MUT) reporter constructs. These constructs were used alongside miRNA mimics for PTEN or mTOR, or their respective negative controls. Post‐transfection, luciferase activity was assessed using a dual‐luciferase reporter system (Promega, USA) according to the manufacturer's instructions. The results were examined to determine the interaction levels between the miRNAs and their target genes, with luciferase activity indicating the binding efficiency of the miRNA to its target.

### 2.8 Statistical Analysis

The experimental data were analysed with GraphPad Prism 8 software. All measurements were represented as the mean ± standard deviation (SD) and each experiment was conducted at least three times to ensure accuracy. To determine the significance of differences between two groups, an independent t‐test was applied. For comparisons among three or more groups, a one‐way ANOVA was performed to assess the variance. A *p*‐value below 0.05 was deemed statistically significant, indicating notable differences in the data.

## 3 Results

### 3.1 Participants General Data

This study compared general data characteristics of HCC patients in experimental and control groups. No significant differences were observed in gender distribution, age, BMI, Child‐Pugh classification, TNM staging or ALT and AST levels between the groups. Gender distribution showed 60% males in the experimental group and 55% in the control group (*p* = 0.565). Average ages were 65.4 and 64.8 years for the experimental and control groups, respectively (*p* = 0.750). Mean BMI values were 26.5 kg/m^2^ and 26.9 kg/m^2^ for the experimental and control groups (*p* = 0.594). Child‐Pugh classification showed 65% grade A and 35% grade B in the experimental group, compared to 60% grade A and 40% grade B in the control group (*p* = 0.543). TNM staging distribution was similar between groups (*p* = 0.784). Biochemical indicators revealed comparable ALT levels: 61.3 U/L in the experimental group and 61.8 U/L in the control group (*p* = 0.886). AST levels were also similar: 56.2 U/L in the experimental group and 55.6 U/L in the control group (*p* = 0.817). In conclusion, no significant differences were found in baseline characteristics between the two groups, indicating their comparability for further analysis.

### 3.2 Histopathological Examination Results

**FIGURE 1:**
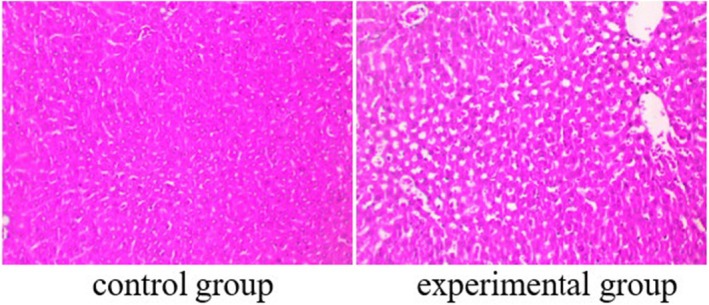
Histopathological examination of liver tumour tissues in HCC patients was conducted using HE staining.

Assessment of Pathological Conditions in Liver Tumour Tissues In this study, we utilised haematoxylin and eosin (HE) staining to evaluate the pathological conditions of liver tumour tissues obtained from patients with HCC. The liver tissues subjected to IOCS intervention exhibited significantly improved pathological features compared to the control group. The HE staining revealed a notable reduction in necrotic areas and a more organised tissue architecture. Cellular morphology showed less pleomorphism, indicating better preservation of liver structure. The HE‐stained liver tissues displayed significant signs of damage, including extensive necrosis, cellular disorganisation and increased inflammatory infiltrates (shown in Figure 1).

### 3.3 Immunofluorescence Results for PTEN Expression

**FIGURE 2:**
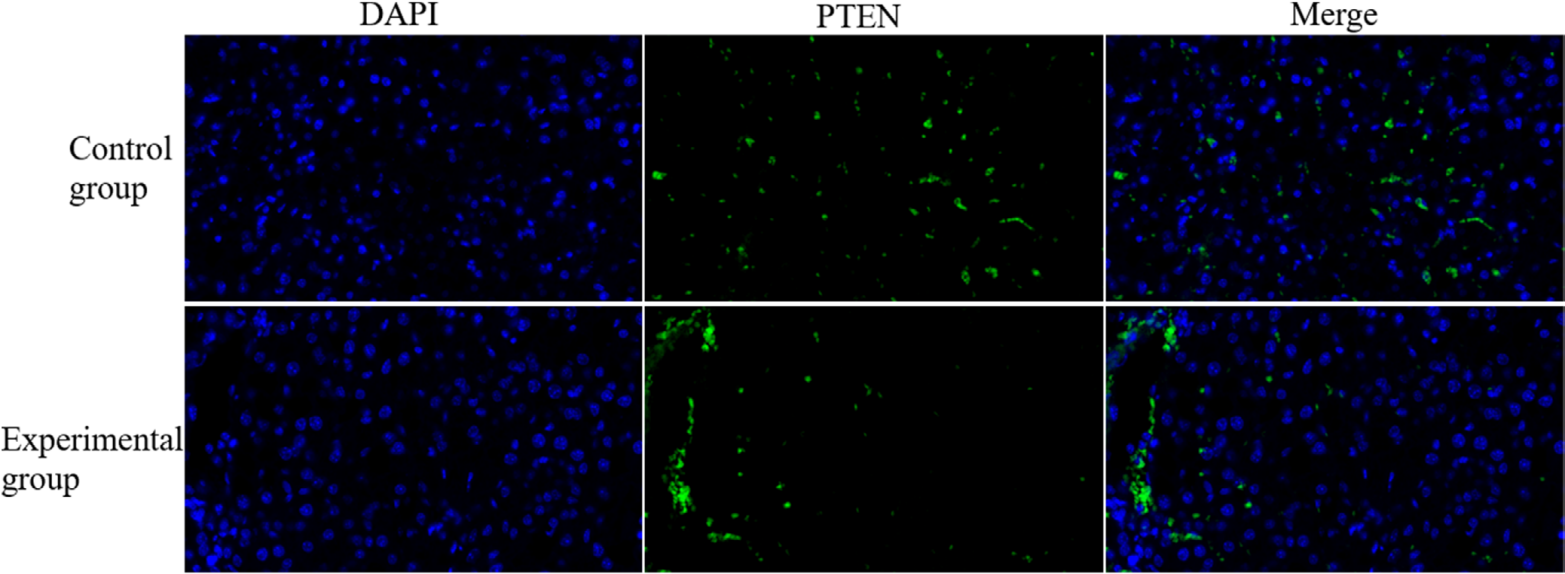
The effect of PTEN in HCC patients was detected using immunofluorescence assay.

In our study, we evaluate the expression of PTEN in liver tumour tissues from patients undergoing HCC treatment. The analysis revealed a significant increase in the number of PTEN‐positive cells in the liver tumour tissues of the experimental group, which underwent IOCS intervention, compared to the control group. Immunofluorescence staining demonstrated a pronounced increase in PTEN expression, with a higher density of PTEN‐positive cells observed throughout the tumour tissue (shown in Figure 2). The control group exhibited markedly fewer PTEN‐positive cells, indicating a reduced expression of PTEN in the tumour microenvironment. These findings highlight the efficacy of IOCS intervention in promoting PTEN expression in liver tumours, which may contribute to improved therapeutic outcomes in HCC patients.

### 3.4 Flow Cytometry Results of Apoptosis Levels in HCC Cells

**FIGURE 3:**
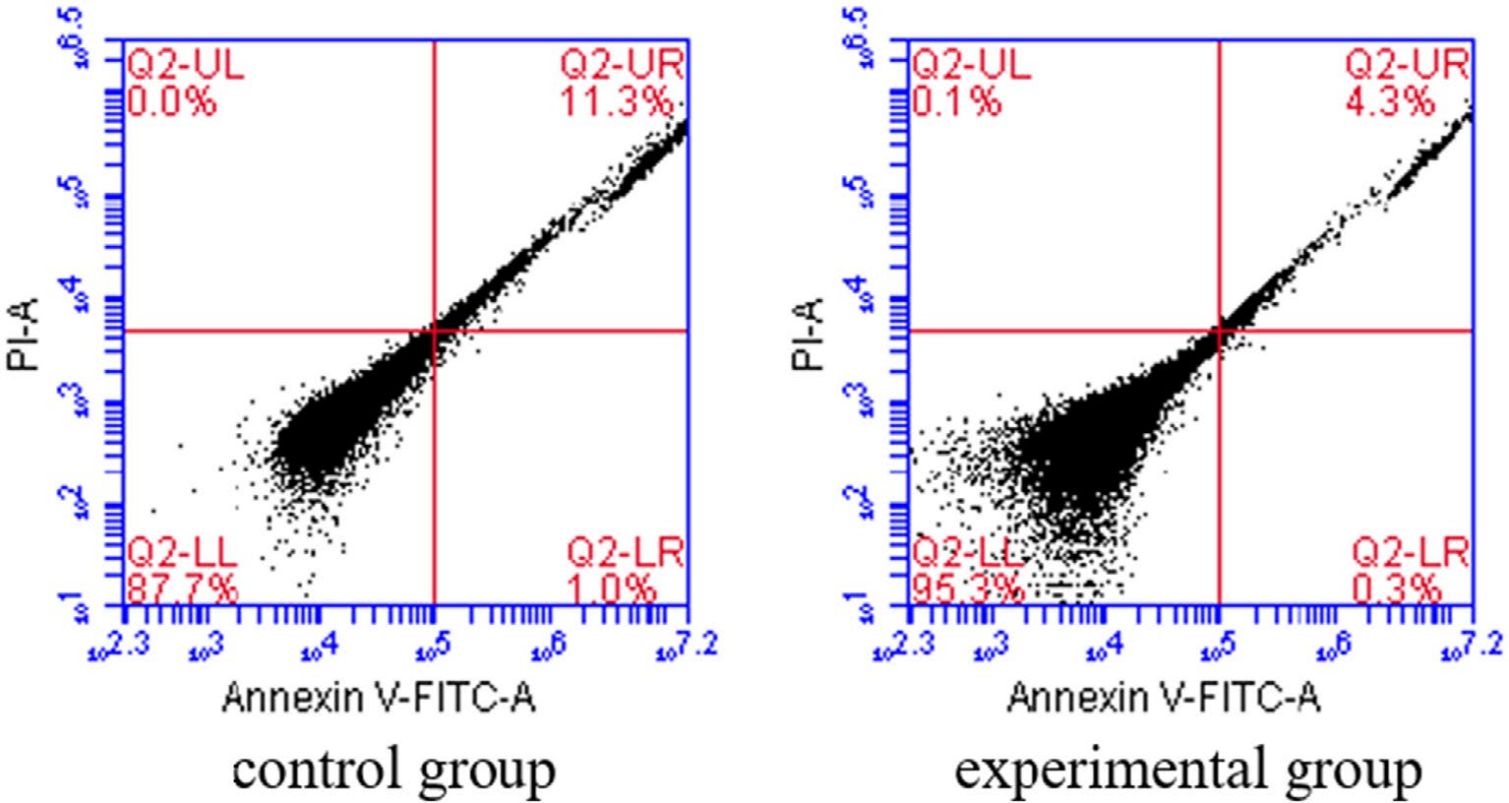
The apoptosis levels in HCC patients from different groups were detected using Flow cytometry assays.

In our study, we employed flow cytometry to assess the apoptosis levels in HCC cells (shown in Figure 3). The experimental group, which underwent IOCS intervention, was compared to the control group that did not receive the IOCS intervention. The flow cytometry analysis indicated a significant reduction in apoptosis levels in the HCC cells from the experimental group. Specifically, the proportion of cells undergoing apoptosis in the experimental group was significantly less than that observed in the control group. Statistical analysis confirmed these findings, with the difference being significant (*p* < 0.05), highlighting IOCS as a beneficial intervention in reducing cell apoptosis, which could have significant implications for therapeutic strategies in HCC. Further studies are warranted to explore the long‐term effects of IOCS on HCC cell survival and overall patient outcomes.

### 3.5 Western Blot Analysis of Protein Expression in HCC Patients

**FIGURE 4:**
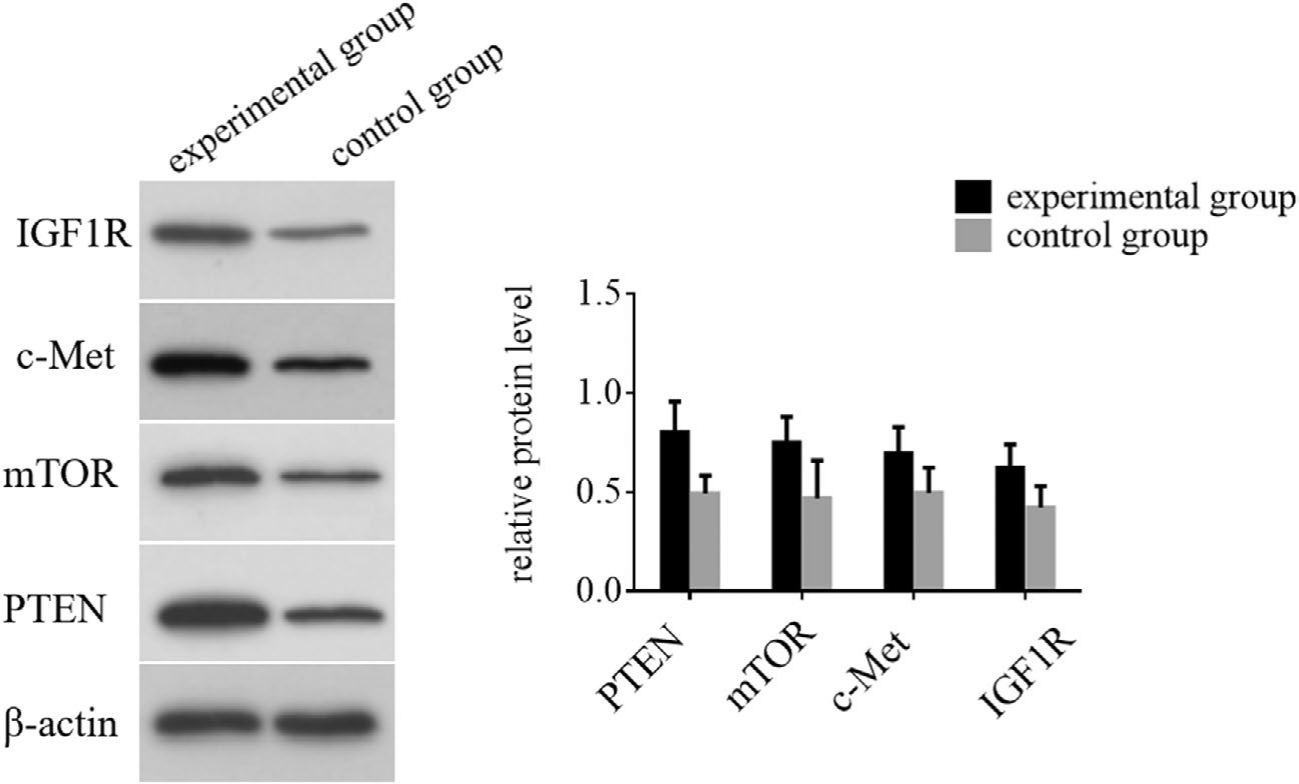
The expression levels of PTEN, mTOR, c‐Met and IGF1R proteins in HCC patients from different groups were determined using Western blotting.

This study evaluates the expression levels of PTEN, mTOR, c‐Met and IGF1R proteins in HCC patients across different groups (shown in Figure 4). Our analysis revealed significant differences in protein expression among the groups. Statistical evaluation validated the importance of these variations, as all measured proteins had *p*‐values below 0.05, indicating that the IOCS intervention significantly affected protein expression in HCC patients. These findings suggest a potential mechanism by which the experimental treatment may influence tumour biology and progression in HCC.

### 3.6 qRT‐PCR Results of miRNA and mRNA Expression in HCC Patients

**FIGURE 5:**
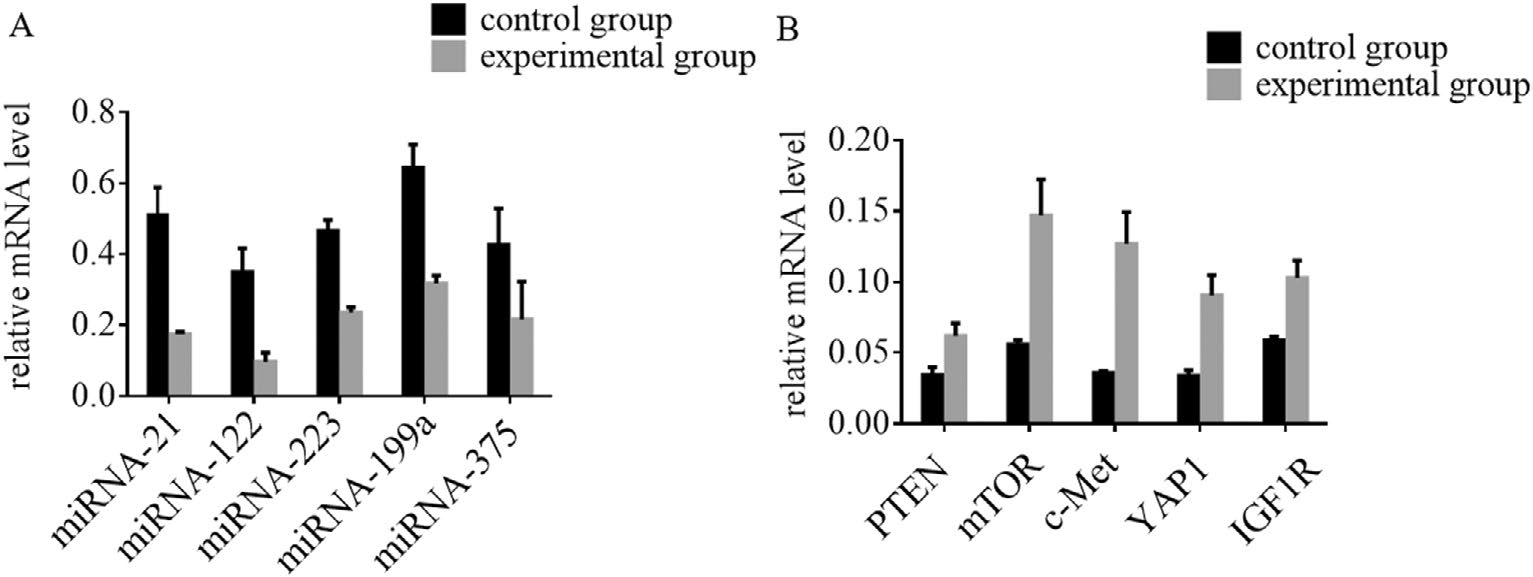
The expression levels of miRNA‐223, miRNA‐21, miRNA‐199a, miRNA‐122 and miRNA‐375, as well as PTEN, mTOR, c‐Met, YAP1 and IGF1R mRNA in HCC patients were determined using qRT‐PCR.

We conducted qRT‐PCR analysis (Figure 5A) to compare the results between the experimental group, which underwent IOCS intervention, and the control group. Our results revealed a statistically significant reduction in the expression levels of miRNA‐21, miRNA‐122, miRNA‐223, miRNA‐199a and miRNA‐375 in the experimental group when compared to the control group, with *p*‐values less than 0.05. This indicates that IOCS intervention had a marked effect on downregulating these miRNAs. In contrast, the mRNA levels of PTEN, mTOR, c‐Met, YAP1 and IGF1R were significantly upregulated in the experimental group (*p* < 0.05) compared to the control group, as illustrated in Figure 5B. This suggests that IOCS intervention promotes the expression of these mRNA targets in HCC patients. These results suggest that the IOCS intervention significantly downregulated the expression of specific miRNAs while concurrently upregulating key mRNA levels associated with tumour suppression and cellular signalling pathways.

### 3.7 Dual‐Luciferase Reporter Gene Assays for miRNA Target Binding

**FIGURE 6:**
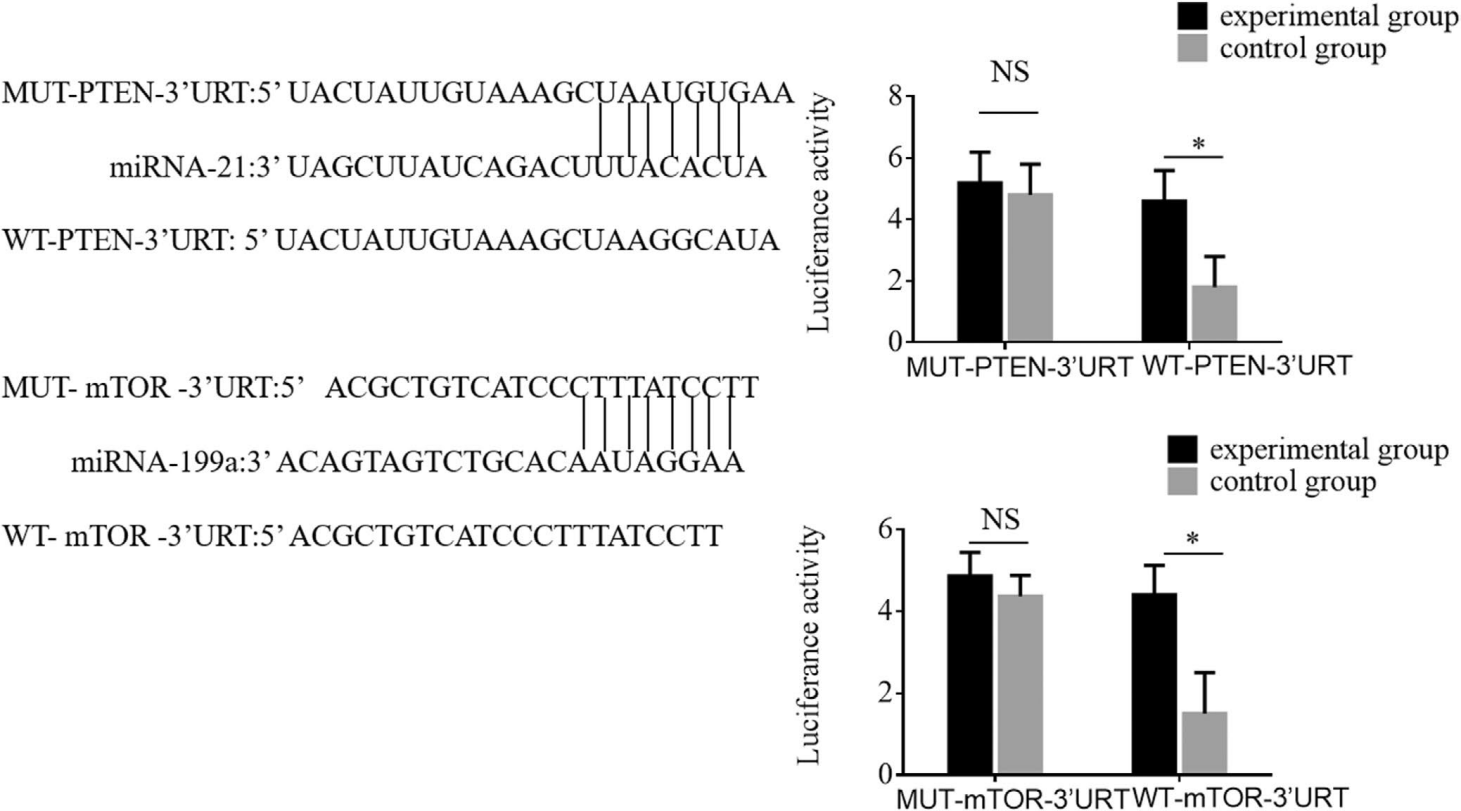
Dual‐luciferase reporter gene assays were performed to confirm that miRNA‐21 specifically inhibits PTEN and that miRNA‐199a specifically inhibits mTOR.

To investigate the specific binding and targeting effects of miRNAs on their respective mRNA targets, we performed dual‐luciferase reporter gene assays focusing on miRNA‐21 and miRNA‐199a. The results confirmed that miRNA‐21 specifically binds to and inhibits PTEN, while miRNA‐199a specifically targets and inhibits mTOR. For PTEN targeting by miRNA‐21, the firefly and sea cucumber luciferase assays yielded the following results: For the mutated (MUT) PTEN 3′ UTR: Firefly Luciferase: 14,864; Sea Cucumber Luciferase: 3525; Ratio: 4.217 Firefly Luciferase: 16,571; Sea Cucumber Luciferase: 3130; Ratio: 5.294 Firefly Luciferase: 17,865; Sea Cucumber Luciferase: 3609; Ratio: 4.951 For the wild‐type (WT) PTEN 3′ UTR: Firefly Luciferase: 17,991; Sea Cucumber Luciferase: 3404; Ratio: 5.286 Firefly Luciferase: 17,877; Sea Cucumber Luciferase: 2829; Ratio: 6.320 Firefly Luciferase: 16,980; Sea Cucumber Luciferase: 3254; Ratio: 5.218 Similarly, miRNA‐199a was shown to specifically bind to and inhibit mTOR, with results indicating significant differences in luciferase activity ratios (shown in Figure 6). These findings provide strong evidence for the specific regulatory roles of miRNA‐21 and miRNA‐199a in the modulation of PTEN and mTOR, respectively, further elucidating the molecular mechanisms of miRNA‐mediated inhibition in HCC.

## 4 Discussion

HCC presents significant challenges in terms of treatment and management, primarily due to its complex biology and the limitations of current therapeutic strategies. Our study aimed to explore the potential of IOCS as a beneficial intervention during the surgical management of HCC [20]. The findings indicate that IOCS may enhance patient outcomes by improving liver tissue pathology and modulating critical signalling pathways.

Our study demonstrates that IOCS has a significant impact on liver tumour pathology in patients with HCC, particularly through the enhancement of PTEN‐positive cell populations [21]. The elevation of PTEN expression suggests that IOCS may foster an environment conducive to tumour suppression [20]. This finding aligns with the literature, such as the work by Ma et al., which identifies PTEN as a potential biomarker and therapeutic target in HBV‐related HCC [22]. which identifies PTEN as a potential biomarker and therapeutic target in HBV‐related HCC. By promoting PTEN activity, IOCS may not only inhibit tumour progression but also improve the efficacy of existing treatments, addressing concerns regarding drug resistance highlighted by Akula et al. [23] Additionally, our results underscore the intricate role of microRNAs (miRs) in regulating gene expression within the PI3K/Akt signalling pathway. The downregulation of specific miRs in the IOCS group indicates a shift in the molecular landscape that may enhance cellular stability and decrease tumour aggressiveness. Recent studies suggest that targeting these miRs could yield synergistic effects when combined with conventional therapies, as multiple signalling pathways often become deregulated in HCC due to various genetic mutations [24]. This multifaceted approach could lead to more effective treatment strategies, a direction supported by growing evidence in the field [5]. The advantages of our research lie not only in the identification of potential biomarkers but also in the therapeutic implications of IOCS in surgical settings [25]. Unlike traditional transfusion methods, which may contribute to immunomodulation and complicate recovery, IOCS allows for the reinfusion of autologous blood, thereby potentially minimising the risks associated with allogeneic transfusions [26]. This aspect could lead to improved haemodynamic stability and reduced postoperative complications, further emphasising the importance of integrating IOCS into routine surgical practices for HCC [26]. However, it is essential to acknowledge certain limitations in our study. The sample size, while adequate for preliminary findings, may not fully capture the heterogeneity of HCC. Future research with larger cohorts is necessary to validate our findings and to explore the potential of IOCS in diverse patient populations [27]. Additionally, while our study highlights significant molecular changes associated with IOCS, further investigations are warranted to elucidate the precise mechanisms by which these changes influence tumour biology and patient outcomes. Moreover, our analysis revealed increased expression levels of mTOR, c‐Met and IGF1R proteins in the experimental group [17]. These proteins are implicated in several critical cellular processes, including growth, survival and metabolism [28]. The observed upregulation of these proteins suggests that IOCS not only aids in cellular recovery but may also foster a more favourable microenvironment for liver tissue regeneration post‐surgery. The integration of IOCS into surgical practices could thus represent a significant advancement in managing HCC, potentially leading to improved surgical outcomes and patient survival.

Our findings highlight the significant role of IOCS in modulating the molecular landscape of HCC. Specifically, we observed a marked reduction in key microRNAs (miRNAs)—including miRNA‐21, miRNA‐122, miRNA‐223, miRNA‐199a and miRNA‐375—in the IOCS group. Badami et al. [29] noted that dysregulation of miRNAs in HCC can contribute to disease progression; our results suggest that IOCS may mitigate this dysregulation, potentially shifting the tumour phenotype towards a less aggressive state [30]. This alteration in miRNA expression can enhance the efficacy of adjunctive therapies, thereby lowering recurrence rates, a critical consideration in HCC management. The downregulation of these miRNAs indicates a promising therapeutic avenue. Previous studies have implicated these miRNAs in processes such as proliferation and apoptosis, underscoring their relevance in tumour biology. By restoring a more favourable miRNA profile, IOCS may not only support tumour suppression but also improve patient outcomes through enhanced responsiveness to treatment. Additionally, our study supports the notion that IOCS can provide a safer alternative to traditional blood transfusion methods, thereby reducing complications associated with allogeneic transfusions. This aspect positions IOCS as a valuable intervention in surgical oncology, aligning with current trends emphasising personalised and less invasive treatment modalities. While our results are promising, it is important to acknowledge limitations such as the need for larger sample sizes to validate our findings and the necessity for longitudinal studies to assess long‐term outcomes. Future research should further explore the interplay between miRNA dynamics and therapeutic efficacy in HCC, aiming to optimise treatment strategies. Our study contributes to the growing body of literature supporting the role of IOCS in surgical oncology. However, further investigations are warranted to fully elucidate the mechanisms by which IOCS influences tumour biology and to identify additional safety biomarkers that could enhance the application of autologous blood transfusion in HCC patients. Longitudinal studies are also essential to assess the long‐term outcomes associated with IOCS use in this patient population.

While this study provides valuable insights into the effects of IOCS in HCC patients, several limitations should be acknowledged. The sample size of 80 patients may limit the statistical power and generalisability of the findings. Additionally, the study was conducted at a single institution, which may introduce potential biases. The long‐term impact of IOCS on patient outcomes, including survival rates, was not assessed. Further multi‐centre studies with larger cohorts and long‐term follow‐up are needed to confirm these findings.

Recommendation and future perspectives: Given the promising results observed in this study, we recommend conducting larger‐scale, multi‐centre trials to validate the impact of IOCS on HCC patients. Future research should focus on exploring the long‐term effects of IOCS on survival rates, recurrence and overall patient prognosis. Additionally, investigating the molecular pathways modulated by IOCS will provide further insights into its mechanisms of action, helping to refine its use as a therapeutic strategy. Incorporating a broader range of patient demographics and treatment protocols will also enhance the generalisability of these findings. Furthermore, repurposing existing drugs and administering prophylactic treatments, such as vitamin D, with modulatory effects, may also offer a promising approach to improving outcomes in HCC patients [31].

In conclusion, our findings suggest that IOCS has the potential to play a pivotal role in the surgical management of HCC by not only improving immediate postoperative recovery but also by influencing tumour biology favourably. By reducing cell apoptosis and promoting tumour‐suppressive mechanisms, IOCS may offer a novel therapeutic strategy in the management of HCC, ultimately leading to better patient outcomes. Further research is essential to confirm these findings and explore their clinical implications.

## Author Contributions

**Yong Cheng:** conceptualization (equal), data curation (equal), formal analysis (equal), investigation (equal), methodology (equal), resources (equal), software (equal), supervision (equal), validation (equal), visualization (equal), writing – original draft (equal), writing – review and editing (equal). **Yue Wang:** conceptualization (equal), data curation (equal), formal analysis (equal), investigation (equal), methodology (equal), resources (equal), software (equal), supervision (equal), validation (equal), visualization (equal), writing – original draft (equal), writing – review and editing (equal). **Xiao‐fang Zhou:** data curation (equal), formal analysis (equal), investigation (equal), methodology (equal), software (equal), supervision (equal), validation (equal), visualization (equal), writing – original draft (equal), writing – review and editing (equal). **Lai‐wei You:** data curation (equal), formal analysis (equal), investigation (equal), supervision (equal), visualization (equal), writing – review and editing (equal). **Xiao‐yi Xie:** data curation (equal), formal analysis (equal), investigation (equal), supervision (equal), visualization (equal), writing – review and editing (equal). **Hao Li:** formal analysis (equal), investigation (equal), supervision (equal), visualization (equal), writing – review and editing (equal). **Mandi Wu:** formal analysis (equal), investigation (equal), supervision (equal), visualization (equal), writing – review and editing (equal). **Jianrong Guo:** funding acquisition (equal), investigation (equal), project administration (equal), supervision (equal), visualization (equal), writing – review and editing (equal).

## Ethics Statement

Ethical approval was given by Gongli Hospital of Shanghai Pudong New Area.

## Consent

Written informed consent was obtained from all patients. All of the authors have Consented to publish this research.

## Conflicts of Interest

The authors declare no conflicts of interest.

## Data Availability

The data are freely accessible upon request.
